# Quantitative Trait Locus (QTL) Mapping Reveals a Role for Unstudied Genes in *Aspergillus* Virulence

**DOI:** 10.1371/journal.pone.0019325

**Published:** 2011-04-29

**Authors:** Julian K. Christians, Manjinder S. Cheema, Ismael A. Vergara, Cortney A. Watt, Linda J. Pinto, Nansheng Chen, Margo M. Moore

**Affiliations:** 1 Department of Biological Sciences and Simon Fraser University, Burnaby, British Columbia, Canada; 2 Department of Molecular Biology and Biochemistry, Simon Fraser University, Burnaby, British Columbia, Canada; David Geffen School of Medicine at University of California Los Angeles, United States of America

## Abstract

Infections caused by the fungus *Aspergillus* are a major cause of morbidity and mortality in immunocompromised populations. To identify genes required for virulence that could be used as targets for novel treatments, we mapped quantitative trait loci (QTL) affecting virulence in the progeny of a cross between two strains of *A. nidulans* (FGSC strains A4 and A91). We genotyped 61 progeny at 739 single nucleotide polymorphisms (SNP) spread throughout the genome, and constructed a linkage map that was largely consistent with the genomic sequence, with the exception of one potential inversion of ∼527 kb on Chromosome V. The estimated genome size was 3705 cM and the average intermarker spacing was 5.0 cM. The average ratio of physical distance to genetic distance was 8.1 kb/cM, which is similar to previous estimates, and variation in recombination rate was significantly positively correlated with GC content, a pattern seen in other taxa. To map QTL affecting virulence, we measured the ability of each progeny strain to kill model hosts, larvae of the wax moth *Galleria mellonella*. We detected three QTL affecting *in vivo* virulence that were distinct from QTL affecting *in vitro* growth, and mapped the virulence QTL to regions containing 7–24 genes, excluding genes with no sequence variation between the parental strains and genes with only synonymous SNPs. None of the genes in our QTL target regions have been previously associated with virulence in *Aspergillus*, and almost half of these genes are currently annotated as “hypothetical”. This study is the first to map QTL affecting the virulence of a fungal pathogen in an animal host, and our results illustrate the power of this approach to identify a short list of unknown genes for further investigation.

## Introduction

*Aspergillus* is a genus of ubiquitous fungi that typically grow on decaying organic matter [1] but can also cause life-threatening infections in immunocompromised patients. For example, *Aspergillus* infections are responsible for approximately 9–17% of deaths in the first year following transplantation among liver, heart and lung transplant recipients [2], [3]. Even with treatment, systemic infections are associated with mortality rates between 30–90%, depending on the patient group [4], [5], underlining the need for new antifungal agents. Currently, most antimicrobial drugs block basic functions of pathogens rather than targeting specific virulence traits [6]; therefore, understanding the genes that contribute to virulence could facilitate the identification and targeting of pathogen-specific pathways.

The virulence of *Aspergillus* species is determined by multiple factors that include the ability to acquire iron, grow at mammalian body temperature, and adhere to the host respiratory epithelium, as well as the ability to produce mycotoxins, conidial pigments and melanin [4], [5], [7], [8], [9], [10], [11]. Given this trait complexity, and natural variation in virulence-related traits within *Aspergillus* species [12], [13], [14], [15], it should be possible to identify virulence-related genes using quantitative trait locus (QTL) mapping. QTL are genomic regions that contribute to variation in complex traits such as virulence and are identified through association between genetic markers and phenotype. Because QTL mapping uses molecular markers, this approach is unbiased and can identify genes and/or regulatory regions that are either unknown or not expected to contribute to a given phenotype. Despite an enormous number of QTL studies of animals and plants, there has been relatively little QTL work with fungi. The few studies that have mapped QTL affecting virulence-related traits in fungi have been extremely successful in terms of gene identification. For example, genes contributing to variation in the ability to grow at elevated temperatures among *Saccharomyces cerevisiae* isolates from human patients have been mapped [16], as has a gene affecting virulence traits in the pathogenic yeast, *Cryptococcus neoformans* [17].

Typically, the first step to mapping QTL is to cross two different wild-type strains. Most systemic *Aspergillus* infections are caused by *A. fumigatus* [1] but when we initiated this work a sexual cycle had not yet been observed in this species (it has been demonstrated since [18]). We therefore examined *A. nidulans*, a species that is also responsible for some infections [1], and readily undergoes sexual reproduction. Although responsible for fewer infections than *A. fumigatus*, *A. nidulans* is more resistant to certain antifungal drugs [19], [20], more resistant to human phagocytic defenses *in vitro* [21] and more virulent in patients with chronic granulomatous disease [22] than *A. fumigatus*. In addition to its clinical relevance, for decades, *A. nidulans* has been a model organism for the study of a variety of cellular processes [23].

The objective of the present study was to map QTL affecting virulence in *A. nidulans*. Because *Aspergillus* virulence is determined by multiple factors, we took an unbiased approach to detect variation in virulence regardless of the underlying causes. Specifically, we mapped QTL affecting the ability of *A. nidulans* to kill an animal host. Although infection of immunosuppressed rodents is the model that most closely approximates human disease, QTL mapping requires testing many progeny in replicate, and therefore we used a well-characterized insect host: larvae of the wax moth, *Galleria mellonella*. Although there are major differences between mammalian and insect immune systems, pathogenic fungi often require the same traits for virulence in mammalian and non-vertebrate hosts [24]. Furthermore, some signaling pathways involved in the innate immune response are conserved among insects and mammals, and there are also parallels between phagocytosis by insect hemocytes and by human neutrophils [24]. Correlation between virulence in insect and mammalian models has been observed in *A. fumigatus* [25], [26], [27], *Candida albicans* [28] and *Yersinia pseudotuberculosis* [29]. In addition to our *in vivo* measure of virulence, we also measured growth on solid medium so that we could distinguish between QTL with specific effects on virulence from QTL affecting growth both *in vitro* and *in vivo*. Because iron acquisition and tolerance of low iron conditions are thought to be important virulence factors [30], [31], we measured growth on both low iron and iron-supplemented media.

Mapping QTL also requires a linkage map that describes the distance between loci in terms of how frequently recombination occurs (genetic distance), rather than the number of base pairs (physical distance). Although there is already a linkage map for *A. nidulans*, the existing map is based largely on phenotypic markers [32] and so would not be suitable for mapping in progeny from two wild-type strains. We therefore created a single nucleotide polymorphism (SNP)-based linkage map by genotyping a panel of progeny at SNP throughout the genome.

## Results

### Linkage map

Of the 768 SNPs genotyped, 29 were excluded because they were not polymorphic, or had heterozygous or missing genotypes for many samples, including parent strains (Fungal Genetics Stock Centre A4 and A91), leaving a total of 739 markers. Several progeny had near-identical genotypes (>700 genotypes in common): 7 were identical to A4, 7 were identical to A91, while there were 12 groups of identical progeny genotypes ranging in size from 2–5 strains. There were 61 unique progeny genotypes (not including parental genotypes), and only these genotypes were included in linkage and QTL mapping.

Building linkage groups using all markers and requiring LOD scores of 6 or more and a maximum recombination frequency of 0.2 to establish linkage yielded 30 linkage groups and 3 unlinked loci (cntg-29-52692; cntg-43-189692; cntg-84-580184). Linkage groups and the three unlinked markers were combined to correspond to *A. nidulans* chromosomes on the basis of markers located on separate linkage groups but known to be located on the same contig, and/or contigs located on separate linkage groups known to be located on the same chromosome [33], [34]. In almost all cases where linkage groups were combined in this way, the recombination frequency between adjacent markers was 0.23 or lower, and the support for linkage was a LOD score of 3.7 or higher. However, the recombination frequency between markers cntg-55-265971 and cntg-55-175295 was 0.29, for which the LOD score was 2.22.

Combining linkage groups in this way and using MapDisto to order the markers, we obtained marker orders that were largely consistent with the genome sequence. Where there were discrepancies between the order calculated by MapDisto and that based on the genome sequence, we calculated map length based on the marker order from the genome sequence. In some cases, the genome sequence yielded a shorter map length than the MapDisto distance, and in 9 other cases the genome sequence yielded a map length within 20% of the MapDisto order (considering only the contentious markers and not the entire chromosome), and we adopted the genome order for further analyses. However, in one case the map length based on the genome sequence was substantially longer than that using the MapDisto order. On Chromosome V, the MapDisto order between markers cntg-157-107424 and cntg-98-505170 (Table S1) yielded a map length of 21.3 cM for this region, whereas the marker order from the genome sequence yielded a length of 58.5 cM. This discrepancy was due to an inversion of all of the markers from contigs 88 and 89, and no other markers. It is therefore not clear whether this is an error in the genome assembly in which the order of these two contigs was reversed. This region has been suggested to contain the centromere and has been difficult to map previously [35]. Four markers were on contigs not placed on chromosomes in the current genome sequence: we mapped cntg-185-3866 to Chromosome VII, cntg-202-4715 to Chromosome I, and cntg-221-4469 and cntg-243-2551 to Chromosome II.

We present the linkage map used in subsequent analyses in Table S1, rather than as a figure because of the large number and density of markers. Table 1 summarizes the results of linkage mapping. Chromosomes ranged in size from 331.3 cM (Chromosome IV) to 577.2 cM (Chromosome VII), with an estimated genome size of 3705 cM. The average intermarker spacing per chromosome ranged from 3.8 cM (Chromosome I) to 6.3 cM (Chromosome V).

**Table 1:** Summary of linkage mapping in cross between *A. nidulans* strains A4 and A91.

Chromosome	No. of markers	Average marker spacing (cM)	Genetic length covered by markers (cM)	Genetic length including chromosome ends (cM)	Physical length covered by markers (kb)	Ratio of physical distance to genetic distance (kb/cM)
1	101	3.8	383.2	390.9	3664	9.6
2	106	4.2	439.7	448.1	3986	9.1
3	82	5.3	425.5	436.0	3357	7.9
4	74	4.4	322.5	331.3	2732	8.5
5	77	6.3	480.3	492.9	3071	6.4
6	77	6.1	460.4	472.5	3313	7.2
7	110	5.2	566.8	577.2	4464	7.9
8	112	4.9	546.3	556.2	4825	8.8
Total	739	5.0	3624.7	3705.0	29412	Average 8.1

### Variation in recombination rate is correlated with GC content

**Figure 1:**
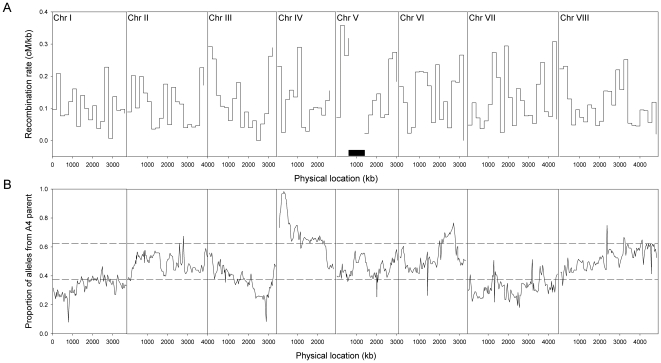
Recombination rate and segregation ratios throughout the genome. (A) Variation in recombination rate averaged over non-overlapping 200 kb windows across Chromosomes I–VIII. Recombination rate is not displayed for the potential inversion on Chromosome V, which is denoted by the black rectangle. (B) Marker segregation ratios across Chromosomes I–VIII. The 95% confidence interval for a 1∶1 ratio is indicated by horizontal dashed lines at 0.375 and 0.625.

**Figure 2:**
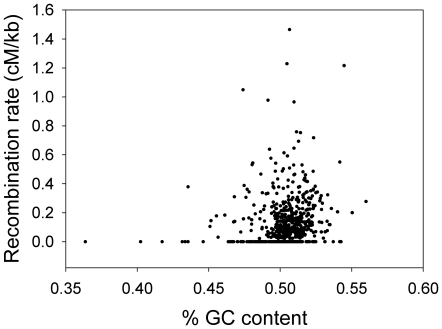
Correlation between the recombination rate (cM/kb) and GC content of 647 intermarker intervals. The number of intermarker intervals is smaller than the number of markers because intervals within the potential inversion on Chromosome V are not included, and four markers are not placed in the current genome assembly.

Our linkage map provided the genetic positions of the markers and the *A. nidulans* genomic sequence [34], [36] provided their physical positions (included in Table S1). The average ratio of physical distance to genetic distance per chromosome varied from 6.4 kb/cM (Chromosome V) to 9.6 kb/cM (Chromosome I), with further variation within chromosomes shown in Fig. 1A. Variation in recombination rate (cM/kb) between intervals was significantly positively correlated with GC content (Spearman rank r = 0.19; N = 647; P<0.0001; Fig. 2); this relationship remained significant when intervals with extreme GC content (<45% or >55%) were removed (Spearman rank r = 0.17; N = 637; P<0.0001). We analyzed the correlation between recombination rate and GC content over different scales by averaging these parameters within non-overlapping windows of various sizes. The relationship remained significant up to a window size of 450 kb (Spearman rank r = 0.33; N = 68; P<0.007), but was not significant at larger scales. We did not include intervals located within the Chromosome V inversion in these analyses in case the inversion affected recombination rates.

### Many markers show skewed segregation ratios

We observed skewed segregation ratios throughout much of the genome: Markers on most of Chromosomes I and VII and approximately half of Chromosome III were significantly skewed towards A91 alleles (not accounting for multiple tests), whereas markers on most of Chromosome IV and part of Chromosome VI were significantly skewed towards A4 alleles (Fig. 1B). Segregation data for all markers are available in Table S1.

### Growth is a quantitative trait

**Figure 3:**
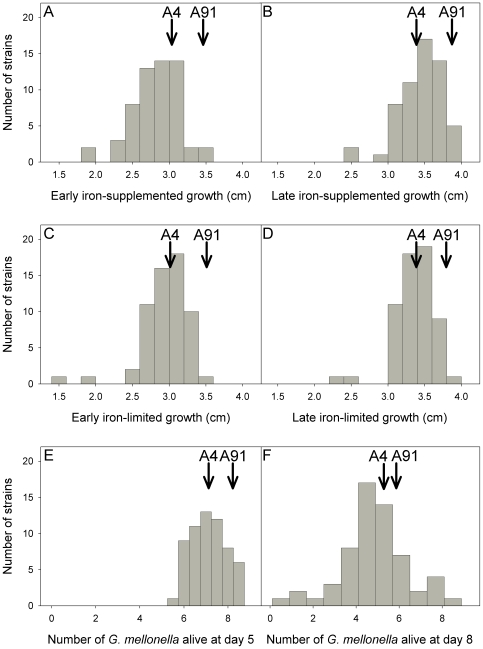
Phenotypic distributions of traits measured in 61 unique progeny genotypes. (A) early iron-supplemented growth, (B) late iron-supplemented growth, (C) early iron-limited growth, (D) late iron-limited growth, and number of *G.mellonella* alive at (E) 5-days post injection and at (F) 8-days post injection.

There was significant variation among strains in early growth (colony diameter at day 3) and late growth (the difference in colony diameter between days 3 and 6) on both iron-limited and iron-supplemented medium (P<0.0001 in all cases). Differences between the parental strains were also significant, with A91 showing more growth than A4 (P<0.0001 in all cases). The distributions of all traits are approximately normal, with only two strains showing very poor growth *in vitro* (Fig. 3). Furthermore, the progeny are distributed asymmetrically around the parental strains (Fig. 3), with few progeny showing higher growth than strain A91.

Surprisingly, early growth was significantly higher on iron-limited (2.97±0.03 cm standard error) compared to iron-supplemented medium (2.85±0.03 cm; paired t_92_ = −5.04; P<0.0001), whereas late growth showed the expected pattern (iron supplemented: 3.48±0.03 cm; iron-limited: 3.38±0.03 cm; paired t_92_ = 4.02; P<0.0001). There was a significant correlation between growth on iron-limited and iron-supplemented medium (early growth: Pearson r_92_ = 0.73, P<0.0001; late growth: Pearson r_92_ = 0.62, P<0.0001).

### In vivo virulence is a quantitative trait

None of the *G. mellonella* larvae from any of the negative controls died. There was little mortality in the first 3 days after injection with *A. nidulans*, therefore we limited analysis to *G. mellonella* survival on days 4 through 8. Variation among strains in the number of *G. mellonella* surviving was significant on days 7 and 8 post-injection (day 7: F_94,270_ = 1.77; P = 0.0002; day 8: F_94,270_ = 1.98; P<0.0001), but not on the other days. However, the difference between the two parental strains was significant on days 4 and 5 (day 4: F_1,270_ = 6.47; P = 0.01; day 5: F_1,269_ = 8.32; P = 0.004). Injection with strain A4 resulted in lower *G. mellonella* survival, i.e., A4 had higher virulence compared to A91. We therefore analyzed the results of survival on days 5 and 8 in further analyses; the distributions of these traits are shown in Fig. 3.

### QTL mapping reveals loci affecting virulence are distinct from those affecting growth

We performed composite interval mapping with various window sizes (5 cM, 10 cM and 20 cM) and regression models (forward, backward, or forward and backward), which yielded similar results. However, increasing the number of background markers increased the statistical support for QTL, and therefore resulted in the identification of a greater number of significant QTL. Because the number of significant QTL was sensitive to the number of background markers, we report results from the forward and backward regression model (probability into, 0.05; probability out, 0.1), in which the number of background markers was determined automatically rather than by user input. Significance thresholds were calculated by permutation and did not make any assumptions about trait distribution.

**Figure 4:**
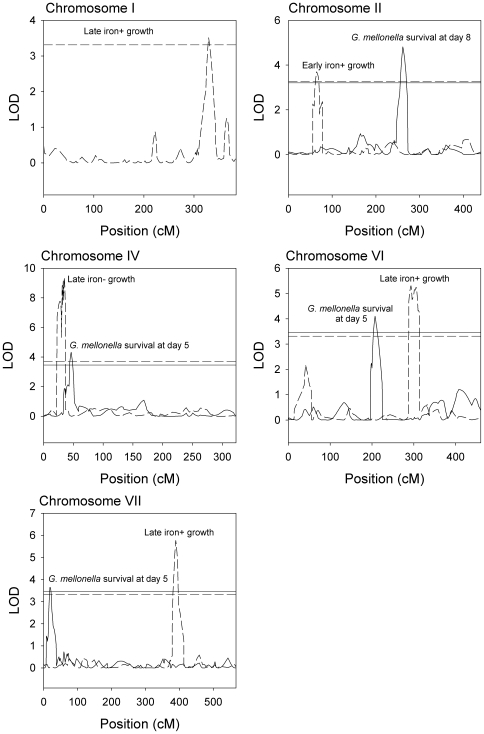
LOD plots from composite interval mapping of growth and virulence for Chromosomes I, II, IV, VI, and VII. Horizontal lines show the genome-wide significance thresholds obtained by permutation. For LOD plots and thresholds, dashed lines denote growth traits and solid lines denote virulence traits. For clarity, we have omitted plots for early and late iron-supplemented growth and early iron-limited growth on Chromosome IV and plots for early iron-supplemented growth on Chromosome VI.

We identified QTL affecting all traits (Table 2). The proximal region of Chromosome IV was associated with variation in all growth traits as well as the number of *G. mellonella* larvae alive at 5 days post-injection (Fig. 4). However, the growth QTL appeared to be distinct from the virulence QTL; there was no overlap in the 2-LOD support interval (Table 2), which is a conservative estimate of the 95% confidence interval [37]. Markers in this region showed extremely skewed segregation ratios with an excess of A4 alleles (Fig. 1B). At the estimated location of the growth QTL, only 2–4 strains carry the A91 allele, making the support for this QTL somewhat suspect. However, the estimated effects of the QTL are consistent with effects of selection on this locus; the A4 allele increases growth, and so the growth QTL may have caused the skewed marker ratio.

**Table 2:** Summary of QTL positions and effects.

Trait	Chromosome	Estimated position (cM)	Estimated effect size^a^	LOD score at peak	% variance explained by QTL	2-LOD support interval (cM)	Proximal marker flanking 2-LOD interval (position in kb)	Distal marker flanking 2-LOD interval (position in kb)
*G.mellonella* survival								
Day 5	IV	47	−0.55	4.32	19	43–51	cntg-126-39521 (420.7)	cntg-124-54688 (492.1)
	VI	207	0.40	4.10	18	201–221	cntg-53-38593 (1416.8)	cntg-52-2303 (1534.7)
	VII	19	0.35	3.65	17	14–32	cntg-167-30076 (253.1)	cntg-165-13625 (433.7)
Day 8	II	262	0.70	4.82	20	255–271	cntg-65-149896 (2291.3)	cntg-65-55949 (2385.3)
Growth								
Early, iron-supplemented	II	65	0.12	3.70	12	56–71	cntg-135-275672 (354.4)	cntg-138-30822 (484.5)
	IV	35	0.45	5.45	26	34–37	cntg-127-64936 (354.4)	cntg-127-8816 (410.5)
	VI	296	0.13	4.32	14	286–301	cntg-51-684909 (1966.4)	cntg-51-460598 (2190.8)
Late, iron-supplemented	I	329	-0.11	3.51	11	319–341	cntg-112-222346 (3030.3)	cntg-113-57473 (3219.3)
	IV	35	0.44	6.92	30	34–37	cntg-127-64936 (354.4)	cntg-127-8816 (410.5)
	VI	293	0.14	5.31	19	288–313	cntg-51-684909 (1966.4)	cntg-51-372274 (2279.1)
	VII	390	-0.14	5.77	20	383–396	cntg-36-61980 (3247.2)	cntg-38-171201 (3530.6)
Early, iron-limited	IV	35	0.59	13.72	44	34–37	cntg-127-64936 (354.4)	cntg-127-8816 (410.5)
Late, iron-limited	IV	35	0.46	9.39	38	34–37	cntg-127-64936 (354.4)	cntg-127-8816 (410.5)

a A positive effect size indicates that the A4 allele increases the value of the trait compared to the A91 allele and vice versa. Effect sizes are in the units of the trait (i.e., number of *G. mellonella* larvae in the case of virulence and cm in the case of growth).

Elsewhere in the genome, QTL affecting virulence were distinct from QTL affecting growth. In addition to the Chromosome IV QTL, two other QTL affecting the number of *G. mellonella* larvae alive at 5 days post-injection were detected on Chromosomes VI and VII, and one QTL affecting the number of larvae alive at 8 days post-injection was detected on Chromosome II.

### Virulence QTL regions contain no known candidate genes

The markers flanking the 2-LOD support intervals of the virulence QTL on Chromosomes II, VI, VII span regions of 93.9, 117.9 and 180.6 kb, respectively (Table 2). However, we were able to exclude large parts of these regions where parental strains A4 and A91 share identical sequence since regions without sequence variation cannot be responsible for the effects of QTL; a gene may be important for virulence, but if there is no sequence variation in that gene between the parental strains, it will not contribute to quantitative variation among progeny strains. Excluding genes that are not within 100 bp of a SNP or other sequence variation, or which harbor synonymous SNP only, the QTL on Chromosomes II, VI, VII contain 7, 16 and 24 genes, respectively [38], which are listed in Table 3. Almost half (20/47) of these genes are annotated as “conserved hypothetical protein”, and to our knowledge none have previously been associated with virulence.

**Table 3:** Genes within virulence QTL regions, excluding genes with no sequence variation or with synonymous SNP only. No sequence variation other than SNP (e.g., indels) was detected in these genes.

Locus	Gene description	Number of non-synonymous SNP a	Other SNP b
Chromosome II			
ANID_03982	conserved hypothetical protein (calcineurin-like phosphoesterase)^c^	2 conservative	0
ANID_03985	MFS transporter	2 radical^d^	0
ANID_03988	conserved hypothetical protein	2 conservative, 3 radical	1 intronic
ANID_03995	delta-aminolevulinic acid dehydratase	0	1 intronic
ANID_03998	conserved hypothetical protein	2 conservative, 2 radical	1 upstream, 3 downstream
ANID_04005	conserved hypothetical protein	4 conservative, 2 radical	2 intronic, 3 downstream
ANID_04006	conserved hypothetical protein (GMC oxidoreductase)^c^	6 conservative	1 upstream, 4 intronic
Chromosome VI			
ANID_03176	ATP-dependent rRNA helicase spb4	0	1 intronic
ANID_03178	deacetylase complex subunit Sds3	1 conservative	0
ANID_03179	conserved hypothetical protein	1 radical	0
ANID_03184	aldose 1-epimerase	0	1 downstream
ANID_03186	conserved hypothetical protein (XPG-I and XPG-N terminal domains)^c^	1 conservative	0
ANID_03193	conserved hypothetical protein	0	1 UTR
ANID_03196	glycosyl hydrolase family 88 protein	0	1 downstream
ANID_03200	glycoside hydrolase family 2 protein	1 conservative, 1 radical	1 upstream,1 intronic, 3 UTR, 1 downstream
ANID_03201	beta-galactosidase	3 conservative, 1 radical	2 upstream, 2 downstream
ANID_03204	MFS alpha-glucoside transporter	1 radical	0
ANID_03205	aldehyde dehydrogenase	3 conservative	1 upstream, 2 intronic,1 downstream
ANID_03209	high affinity copper transporter	1 conservative	0
ANID_10380	dicer-like protein 2	1 radical	0
ANID_10383	conserved hypothetical protein (glycosyl hydrolase family 2, sugar binding domain)^c^	0	1 upstream
ANID_10384	C6 transcription factor	0	1 downstream
ANID_12377	conserved hypothetical protein	1 radical	0
Chromosome VII			
ANID_08919	cytochrome P450 monooxygenase	1 conservative, 1 radical	1 intronic
ANID_08920	cytochrome b5	0	1 downstream
ANID_08921	Dehydrogenase	0	1 intronic, 1 downstream
ANID_08923	conserved hypothetical protein (heterokaryon incompatibility protein)^c^	0	1 intronic
ANID_08925	conserved hypothetical protein	1 radical	1 upstream
ANID_08926	conserved hypothetical protein	0	2 intronic
ANID_08928	ABC multidrug transporter	1 radical	0
ANID_08931	conserved hypothetical protein	0	1 upstream
ANID_08932	TIM-barrel enzyme family protein	0	1 upstream, 2 downstream
ANID_08933	conserved hypothetical protein	0	1 intronic
ANID_08937	3-oxoacyl-(acyl-carrier-protein) reductase 2	1 conservative	0
ANID_08940	conserved hypothetical protein	1 radical	0
ANID_08941	Na/K ATPase alpha 1 isoform	0	1 intronic
ANID_08945	TAM domain methyltransferase	0	1 intronic
ANID_08951	conserved hypothetical protein	0	?^e^
ANID_08953	alpha-glucosidase B	1 conservative	0
ANID_08957	multidrug resistance-associated protein	2 conservative, 1 radical	0
ANID_08958	conserved hypothetical protein	1 radical^f^	0
ANID_08962	conserved hypothetical protein	?^e^	0
ANID_08968	isoflavone reductase	1 conservative	0
ANID_08970	conserved hypothetical protein	1 conservative	0
ANID_08971	integral membrane protein	0	2 intronic
ANID_11152	1,3-beta-glucanosyltransferase	1 conservative	0
ANID_12385	hypothetical protein	0	1 upstream, 2 intronic

a Non-synonymous SNP were classified as conservative if the BLOSUM80 score was 0 or higher for the substitution, or radical if the BLOSUM80 score was negative [74].

b Other SNP include SNP in introns, untranslated regions (UTR) and within 100 bp upstream or downstream of the transcript. Synonymous SNP in coding regions are not included.

c Protein domains for conserved hypothetical proteins were identified by a BLAST search of the Broad Institute database [38].

d There are 3 SNP, but 2 affect the same codon.

e No clear polymorphism, but ambiguity due to low sequence coverage for strain A91.

f Premature stop codon.

### Early and late growth share one QTL but are also affected by distinct QTL

In addition to the Chromosome IV QTL, we observed QTL affecting iron-supplemented growth on Chromosomes I, II, VI with one QTL affecting both early and late growth, one affecting early but not late growth, and two affecting late but not early growth (Table 2). Apart from the Chromosome IV QTL, no QTL affecting iron-limited growth were detected.

### Variation in spore colour is associated with the wA gene

The parental strains used in this study differed in spore colour, and among the unique progeny genotypes, 32 had the wild-type green colour of strain A4, and 28 had the beige colour of strain A91 (spore colour was not recorded for one strain), a ratio that was not significantly different from 50∶50 (χ^2^ = 0.27, P = 0.61). Single marker analysis revealed almost perfect correspondence between the Chromosome II markers cntg-142-37489and cntg-143-2465 and spore colour, with only one unique genotype having the A4 green colour but carrying the A91 allele. At flanking markers cntg-142-11335 and cntg-143-25286, there were two progeny for which spore colour did not match genotype, indicating that the mutation affecting spore colour is located between these two markers that are located 57.8 kb apart. There are 17 genes within this region including the *wA* gene, in which mutations are known to cause white conidia [39], [40]. The flanking markers cntg-142-11335 and cntg-143-25286 are at 165 cM and 168 cM, respectively, indicating that the colour locus does not overlap with the virulence and growth QTL detected on Chromosome II (Table 2).

## Discussion

### QTL mapping has potential to identify novel virulence genes in Aspergillus

Our study is the first to map QTL affecting the virulence of a fungal pathogen in an animal host. We identified separate QTL affecting different measures of virulence: the number of *G. mellonella* larvae alive at 5 days and 8 days post-inoculation, which may reflect virulence factors that act at different stages of infection. Importantly, we identified QTL that affected virulence but not growth, indicating that the underlying genes are true virulence factors as opposed to genes affecting general vigour.

The three virulence QTL regions contain 7–24 genes, many of which are hypothetical genes that were identified using computational methods but have received no study. *A. fumigatus* orthologues have been identified for many of these genes, suggesting that *A. nidulans* may be a useful model for identifying *A. fumigatus* virulence genes.

To our knowledge, none of the genes in our QTL target regions have been previously associated with virulence in *Aspergillus.* Nevertheless, some are stronger candidates than others. β-glucans of the cell wall are involved in triggering innate immune responses against *A. fumigatus* [41], [42] and deletion of a β-1,3-glucanosyltransferase, GEL2, reduced virulence of *A. fumigatus* in a murine model [43]. However, GEL2 is not orthologous to the β-1,3-glucanosyltransferase in the Chromosome VII QTL region. Deletion of *CaCWH41* or *CaROT2*, encoding α-glucosidase I, and α-glucosidase II catalytic subunit, respectively, attenuated the virulence of the pathogenic yeast, *Candida albicans* in a murine model [44]. However, deletion of α-glucosidase I did not affect the virulence of *A. fumigatus* [45] and none of these genes are orthologous to the α-glucosidase B within the Chromosome VII QTL region.

Four of the candidate genes in our QTL regions are members of the ATP-binding cassette (ABC) and major facilitator superfamily (MFS) families of transporters. The high representation of these families within our target regions is not surprising given that these genes are very common in fungal genomes; there are 45 ABC transporters and 356 MFS transporters in the *A. nidulans* genome [46]. ABC and MFS transporters are thought to contribute to virulence by facilitating the export of mycotoxins from fungal cells, and by removing host defence compounds [46]. Although a number of genes from these families have been implicated in plant pathogenesis, only one ABC transporter has been shown to contribute to fungal virulence in a mammalian host [47]. This *C. albicans* gene, *MLT1*, is not orthologous to either of the ABC transporters in the Chromosome VII QTL region. The lack of obvious candidates and the large proportion of hypothetical genes within our QTL regions illustrate the power of QTL mapping to identify a short list of unknown genes for further investigation.

Our study demonstrates that the effects of some virulence QTL are sufficiently large, and that quantitative variation in virulence can be measured with sufficient precision, that it is possible to map QTL affecting *in vivo* virulence in fungal pathogens. Although we phenotyped and genotyped 94 progeny, genotyping revealed only 61 unique genotypes; the presence of clones among progeny has been previously reported in *Cryptococcus neoformans* [48], [49]. Despite this substantial reduction in sample size, we were still able to map QTL to relatively small regions. A larger sample size would allow still greater resolution, i.e., fewer genes per QTL.

We found QTL affecting virulence even though the difference in this trait between parental strains was very modest. This is not unexpected, since one strain may harbour some alleles that increase virulence, and others that decrease virulence compared with the other strain. We selected these parental strains because they differed in spore colour, which was necessary to identify an outcrossed cleistothecium. Had we used parental strains with a greater difference in virulence, we expect that we would have identified more and/or larger QTL. *A. fumigatus* is heterothallic and shows quantitative variation in virulence related traits [12], [13], [14], [15], including virulence in *G. mellonella* [50], and thus it will be possible to cross strains differing in virulence in this species.

This is the first study to use infection of *G. mellonella* with *A. nidulans* as a model of *Aspergillus* virulence. A previous study injected a much lower inoculum of *A. nidulans* (3000 conidia compared with over 10000 in the present study) into *G. mellonella* but observed no mortality, whereas *A. flavus* was found to be virulent [51]. Although there are parallels between insect and mammalian immune responses [24] and correlation between virulence in insect and mammalian models has been observed [25], [26], [27], there are obviously limitations in extrapolating results from our *G. mellonella* model to human disease due to the adaptive immune response of vertebrates, among other factors. For instance, while some conidial colour mutants of *A. fumigatus* show reduced virulence in mammalian models, colour mutants were found to have increased virulence in *G. mellonella* [52]. Ultimately, virulence genes that we identify through subsequent work must be tested in immunocompromised rodents.

We observed significant variation in virulence between the parental strains and among the progeny, but this variation was subtle. Accurate measurement of small differences in virulence required taking many steps to reduce experimental error: suspensions were based on the number of viable conidia and were double checked for accuracy, the weight and age of larvae were kept within a narrow range, and three replicates were performed. Quantitative genetic variation in radial growth and the production of cleistothecia has previously been documented in a cross between two wild-type isolates of *A. nidulans* [53], but this early work did not examine traits related to virulence.

### QTL affecting growth

Several studies have suggested that *A. fumigatus* is the most common pathogen among *Aspergillus* species because of its rapid growth rate. However, the identification of distinct QTL affecting radial growth and virulence results suggest that loci contributing to variation in growth do not contribute to variation in virulence. Although different QTL would likely be found in *A. fumigatus* or different crosses of *A. nidulans*, our results do not support the hypothesis that growth rate is an important virulence factor.

Distinct QTL affecting early and late growth were detected. Surprisingly, we detected QTL affecting iron-supplemented growth and not iron-limited growth, but not vice-versa. However, given the small number of QTL identified, it is not clear whether this reflects a real difference in genetic architecture between iron-supplemented and iron-limited growth. Although we did not expect early radial growth to be lower on iron-supplemented than iron-limited medium, similar results have been observed in *A. fumigatus* [54], perhaps due to low-level iron toxicity to germinating conidia [55]. Alternatively, iron-limitation may have led to longer but thinner hyphae, such that radial growth but not biomass was increased.

### A. nidulans has low ratio of physical distance to genetic distance, which is correlated with GC content over large scales

Despite the widespread use of *A. nidulans* as a genetic model, this is the first SNP-based linkage map for this species. We estimated the size of the genome to be 3705 cM. Our estimate of the average ratio of physical distance to genetic distance across the genome is 8.1 kb/cM, similar to previous estimates for *A. nidulans* [56], and slightly lower than for a number of other fungi [49]. We observed a positive correlation between recombination rate and GC content. While this pattern is widespread in a variety of taxa, we know of no related studies in fungal species other than in *Saccharomyces cerevisiae* [57]. We observed this relationship at a range of scales from the intervals between markers up to windows of 450 kb. In contrast, in *S. cerevisiae,* Marsolier-Kergoat and Yeramian found that the strength of this relationship decreased substantially between windows of 5 kb and 100 kb [58].

Many markers showed skewed segregation ratios in our cross, with an excess of A4 alleles in some regions and an excess of A91 alleles in others. A number of other fungal linkage mapping studies have also found large proportions of markers with skewed ratios [48]. We suspect that at least some of the skew was due to selection, whereby one allele conferred more rapid growth and/or germination of ascospores, making progeny carrying this allele more likely to be isolated. In particular, there is a substantial skew towards A4 alleles at the proximal end of Chromosome IV, and in this region there is a QTL of which the A4 allele enhances growth. Although the A91 parental strain showed a higher growth rate than A4, other loci may have compensated for the Chromosome IV locus. Furthermore, the distribution of growth among the progeny (Fig. 3) suggests there was epistasis among loci affecting growth. If all the QTL affecting growth acted in an additive manner, we would expect the distribution of progeny values to be symmetrical around the parents.

### Mapping further traits

Our genotyped panel of progeny represents a resource for the entire *Aspergillus* research community, since it is now possible to map QTL on any trait that varies among the strains, without the need for further genotyping. These progeny will be analogous to “recombinant inbred lines” which in other taxa have been recognized as powerful tools for QTL mapping, particularly for the study of genotype by environment interactions [37], [59].

## Materials and Methods

### Mapping population

*A. nidulans* strains A4 and A91 were obtained from the Fungal Genetics Stock Center [60]. A4 is the wild-type strain that has been sequenced [23], while A91 is a spore-colour mutant obtained by ultraviolet irradiation of a different wild-type environmental isolate [61]. It was necessary to use a spore-colour mutant to identify a cleistothecium produced by crossing the two strains; *A. nidulans* is homothallic, and genetically distinct strains are much more likely to self than to cross fertilize [62], [63]. Crosses were conducted between A4 and A91 on MYPD agar (3 g malt extract, 3 g yeast extract, 5 g peptone, 10 g glucose and 18 g agar in 1L) at 30°C and cleistothecia were screened for out-crossing by plating ascospores from a single cleistothecium on Neiland's agar plates (described below) and looking for colonies with different pigmentation. Over 100 cleistothecia were screened, but only one was found to be out-crossed, which provided the ascospores for the mapping population described below.

### Marker discovery and genotyping

Mycelia grown on half-strength liquid MYPD medium for 24 to 30 hours at 37°C were harvested by filtration and DNA was extracted using an Epicentre MasterPure™ Yeast DNA Purification Kit [64]. A91 DNA was sent to the Genome Sciences Centre at the British Columbia Cancer Agency (Vancouver, Canada) for library construction and paired-end tag sequencing on the Illumina Genome Analyzer.

A91 sequences were aligned against the reference *A. nidulans* genome sequence [23], [65], obtained from the Broad Institute [38] using MAQ v 0.7.1 [66] with default parameters, except for the maximum outer distance for a correct pair (-a, set to 1500) and the maximum number of mismatched that can always be found (-n, set to 3). Pairs with identical outer coordinates were removed using rmdup, following the suggestion in the MAQ manual for accurate SNP calling. The published genome sequence is based on strain A4, and so differences between the A91 and reference sequences allowed us to identify SNP markers for our population. We selected 768 SNPs spread across the genome for which we had at least 20 times coverage for A91, and at least 90% of the A91 sequence reads supported the presence of a SNP (Table S2). DNA samples from 94 progeny strains, as well as A4 and A91 were sent to the Centre for Applied Genomics at The Hospital for Sick Children (Toronto, Canada) for genotyping of SNPs using the Illumina GoldenGate® Assay.

### Linkage map construction

The construction of a genetic map, which describes the distance between loci in terms of how frequently recombination occurs, was performed using MapDisto version 1.7.0 [67] considering our population to be doubled haploid. We used the Haldane mapping function to translate recombination frequency into map distance (centimorgans, cM), since there is no evidence of crossover interference in *A. nidulans* [68]. We initially required LOD (logarithm of odds) scores of 6 or more and a maximum recombination frequency of 0.2 to establish linkage, but subsequently relaxed the criteria to combine linkage groups known to be on the same chromosome (described in Results section). To calculate the total length of each chromosome, we added two times the average intermarker distance for that chromosome to account for chromosome ends [69].

### Growth media

Strains were grown on Neiland's agar at 37°C (18 g of agar, 20 g of sucrose, 1 g of K_2_SO_4_, 3 g of (NH_4_)_2_SO_4_, 1 g of citric acid, 3 g of K_2_HPO_4_, 3 g of K_2_HPO_4_, 810 mg of MgSO_4_·7H_2_O, 2 mg of thiamine hydro-chloride, 962 µg of MnSO_4_, 20 µg of CuSO_4_, 5.5 mg of ZnSO_4_, per liter of solution with pH adjusted to 6.8–7.0) [70]. For measurement of iron-limited growth, traces of iron were removed from glassware by overnight treatment with 5% HCl and thorough rinsing with deionised water prior to media preparation. For measurement of iron-supplemented growth, 1 mg of FeCl_3_ per litre (3.7 µM) was added to the medium.

### Conidia harvesting and preparation

Conidia from 7-day cultures on iron-limited medium were harvested with 0.05% (v/v) Tween 20 (Sigma Chemica Co., St. Louis, USA) in phosphate buffered saline (pH. 7.4) (PBST), and filtered through Miracloth (Calbiochem) to remove hyphae. Harvested conidia were centrifuged, resuspended in PBST, centrifuged again and resuspended in fresh PBST. The concentration of conidia was determined using a haemocytometer (Hausser Scientific, Horsham, PA).

### Radial growth measurements

To obtain isolated colonies, dilute conidial suspensions were inoculated onto either iron-limited or iron-supplemented Neiland's agar. After approximately 24 hours, mats from single germinated conidia were isolated and transferred to the centre of 10 cm Petri dishes, and at least two germinated conidia of each strain were transferred to two different Petri dishes. Plates with isolated colonies were incubated at 37°C and colony diameter was measured every 24 hours from 3 to 6 days after transfer. Three experiments with at least two replicates per experiment were performed for recombinant strains, and conidia were grown and harvested independently for each experiment. Because the parental strains (A4 and A91) were included with each group of strains measured, there are 34 and 21 replicates of each of the parental strains on iron-limited and iron-supplemented growth, respectively. Early growth was defined as colony diameter at day 3 and late growth was defined as the difference in colony diameter between days 3 and 6.

We initially attempted to measure growth in terms of mass in liquid medium, but switched to measuring radial growth because of large variation between replicates. These initial measures of mass showed the same pattern as subsequent measures of radial growth (i.e., A91>A4; data not shown).

### Virulence in G. mellonella larvae

*G. mellonella* larvae were reared on baby mixed cereal (1200 ml) (H.J. Heinz Company, Canada) supplemented with glycerol (119 ml), water (98 ml), sucrose (100 ml) and multi-vitamins (Enfamil, Poly-vi-sol) (1 ml) at 28–30°C with 50–60% relative humidity and a 12L:12D light cycle as described previously [71]. *G. mellonella* larvae 40 to 50 days of age (in their final instar stage) weighing 0.25–0.30 g were selected for injection.

The goal of this study was to examine variation in virulence among strains, and therefore we needed an inoculum that was not so high that all of the larvae died rapidly, but not so low that none of the larvae died. Preliminary work established that injection of 5 µl of 2080 colony forming units (CFU)/µl yielded intermediate mortality rates, and therefore this was used as the inoculum. To determine the concentration of CFU, conidial suspensions were diluted and inoculated on Neiland's solid agar medium plates (5 plates/strain), which were counted after 36 hours. Suspensions containing 2080 CFU/µl were prepared and used for injection. The concentration of viable conidia was re-confirmed by plate counts of the suspensions used for injection.

Conidial suspensions (5 µl of 2080 CFU/µl) were injected into the hemocoel via the last left proleg using a 25 µl Hamilton syringe (part # 7636 – 01 702RN, Hamilton) with an inner barrel diameter of 0.72 mm and 33 gauge removable needle (part # 7762-06, Hamilton). For each strain and each replicate, one strain was injected into 10 larvae. In addition to all of the strains injected on a given day, the two parental strains, A4 and A91, and two negative controls were included: 10 larvae received 5 µl of PBST, and another 10 larvae received no injection. Between injections, the syringe was washed once with 70% ethanol and twice with PBST to avoid cross contamination. Injected larvae were placed in Petri dishes containing pine wood chips, and dishes were left at 37°C in the dark. Mortality was monitored once a day for 8 days. We performed three replicates for each strain and for each replicate, conidia were grown, harvested, and counted independently, and all replicates were injected on different days. Because the parental strains were always included with the strains injected on a given day, there are 32 replicates of A4 and A91.

Conidia of *A. nidulans* killed by heating at 100°C for 1 hour were also injected into wax moth larvae to see if non-viable conidia contributed to virulence. Lack of viability of heat killed conidia was confirmed by plating on solid medium. This negative control was repeated three times.

### QTL mapping

We mapped QTL by testing the association between phenotype (growth or virulence) and the genotypes of SNPs located throughout the genome. We used composite interval mapping, which scans the genome for QTL while using additional markers as cofactors to account for effects of QTL outside the focal interval, increasing the power to detect QTL and the precision with which positions are estimated [37]. Composite interval mapping was performed using Windows QTL Cartographer Version 2.5 [72], with a walk speed of 1 cM. Genome-wide significance thresholds were determined empirically by permuting the marker data [73], using 1000 permutations. Because the significance thresholds are calculated from an empirical distribution of the test statistic under the null hypothesis that there is no QTL, the analysis does not make any assumptions about the distribution of the phenotypic traits (i.e., traits do not have to be normally distributed).

## Supporting Information

Table S1 ***Aspergillus nidulans*** **linkage map, including genetic and physical positions and segregation data for each marker.**
(XLS)

Table S2 **Sequence flanking SNP markers.**
(XLS)
